# BBD Driven Fabrication of Hydroxyapatite Engineered Risedronate Loaded Thiolated Chitosan Nanoparticles and Their In Silico, In Vitro, and Ex Vivo Studies

**DOI:** 10.3390/mi14122182

**Published:** 2023-11-30

**Authors:** Zoya Saifi, Tanya Ralli, Md. Rizwanullah, Meraj Alam, Divya Vohora, Showkat R. Mir, Saima Amin, Sadia Ameen

**Affiliations:** 1 Department of Pharmaceutics, School of Pharmaceutical Education and Research, Jamia Hamdard, New Delhi 110062, India; zoya783@gmail.com (Z.S.); mdrizwanullah54@gmail.com (M.R.);; 2 Department of Pharmacology, School of Pharmaceutical Education and Research, Jamia Hamdard, New Delhi 110062, India; 3 Department of Pharmacognosy and Phytochemistry, School of Pharmaceutical Education and Research, Jamia Hamdard, New Delhi 110062, India; 4 Advanced Materials and Devices Laboratory, Department of Bio-Convergence Sciences, Jeonbuk National University, Advanced Science Campus, Jeonju 56212, Republic of Korea

**Keywords:** risedronate, thiolated chitosan, PEGylated nanoparticles, osteoporosis, hydroxyapatite

## Abstract

Risedronate sodium (RIS) exhibits limited bioavailability and undesirable gastrointestinal effects when administered orally, necessitating the development of an alternative formulation. In this study, mPEG-coated nanoparticles loaded with RIS-HA-TCS were created for osteoporosis treatment. Thiolated chitosan (TCS) was synthesized using chitosan and characterized using DSC and FTIR, with thiol immobilization assessed using Ellman’s reagent. RIS-HA nanoparticles were fabricated and conjugated with synthesized TCS. Fifteen batches of RIS-HA-TCS nanoparticles were designed using the Box–Behnken design process. The nanoparticles were formulated through the ionic gelation procedure, employing tripolyphosphate (TPP) as a crosslinking agent. In silico activity comparison of RIS and RIS-HA-TCS for farnesyl pyrophosphate synthetase enzyme demonstrated a higher binding affinity for RIS. The RIS-HA-TCS nanoparticles exhibited 85.4 ± 2.21% drug entrapment efficiency, a particle size of 252.1 ± 2.44 nm, and a polydispersity index of 0.2 ± 0.01. Further conjugation with mPEG resulted in a particle size of 264.9 ± 1.91 nm, a PDI of 0.120 ± 0.01, and an encapsulation efficiency of 91.1 ± 1.17%. TEM confirmed the spherical particle size of RIS-HA-TCS and RIS-HA-TCS-mPEG. In vitro release studies demonstrated significantly higher release for RIS-HS-TCS-mPEG (95.13 ± 4.64%) compared to RIS-HA-TCS (91.74 ± 5.13%), RIS suspension (56.12 ± 5.19%), and a marketed formulation (74.69 ± 3.98%). Ex vivo gut permeation studies revealed an apparent permeability of 0.5858 × 10^−1^ cm/min for RIS-HA-TCS-mPEG, surpassing RIS-HA-TCS (0.4011 × 10^−4^ cm/min), RIS suspension (0.2005 × 10^−4^ cm/min), and a marketed preparation (0.3401 × 10^−4^ cm/min).

## 1. Introduction

Chronic bone disease that weakens bones and raises the possibility of fractures as people age is called osteoporosis. As predicted in a previous study, there is an evident rise to 62 lakh cases by 2050 from 16 lakh cases in 1990 [1]. Osteoporosis, a disease that reduces bone mass and strength, can be treated using a number of different approaches. However, there are several restrictions and long-term safety concerns associated with the current therapies [2]. Therefore, more research is required to determine the best therapy choice. Most therapeutic strategies for reducing bone loss and avoiding fractures can be grouped into two categories, i.e., anabolic drugs and anti-resorptive drugs [3]. Bisphosphonates, calcitonin, estrogen selective receptor modulators, and monoclonal antibodies, such as denosumab, are all examples of anti-resorptive medications that work by decreasing the activity of osteoclasts to build bone strength. In contrast, anabolic drugs (recombinant human parathyroid hormone, calcitonin, and estrogen) are able to induce the formation of bones and can reverse bone degeneration, which is caused by the progression of osteoporosis [4]. With inhibition of Farnesyl Pyrophosphate Synthase (FPPS), a key enzyme in membrane protein prenylation, as well as osteoclast detachment from bone, BPs disrupt osteoclastic activity. In the end, they cause apoptosis in osteoclasts, which decreases bone resorption. Treatment with alendronate and risedronate (RIS) is regarded as first-line therapy for controlling and preventing osteoporosis in post-menopausal for both men and women [3].

Bone problems, including Paget’s disease and osteoporosis, are treatable using RIS. It can be swallowed, but taking it that way comes with some precautions to prevent esophageal ulcers, such as taking it while standing up and then washing it down with water [5,6]. If taken with food, absorption is even worse. Therefore, it is strongly advised that no meal to be consumed within two hours and thirty minutes before medication. In addition, it belongs to class III of the biopharmaceutics categorization system (high solubility/low permeability) and has a poor oral bioavailability of 1% [7]. Therefore, there is a need for effective drug delivery systems for RIS that increase bioavailability and decrease the likelihood of esophageal pain.

Given its structural similarity to minerals found in dentin or natural bones and its bioactivity, osteoconductive, non-inflammatory, and biocompatibility properties, Hydroxyapatite (HA) is used widely as a biomaterial for bone regeneration [8,9]. As BPs attach to HA, the binding capacity of bone increases. In addition, BPs have the unique ability to prevent the breakdown of hydroxyapatite (HA) already present in the bone [10]. As a result of this property, HA is a trusted conveyance for the delivery of BPs [11]. Due to its biocompatibility and biodegradability, poly(lactide-co-glycolide) (PLGA) is used in drug delivery systems. Drug release characteristics could be altered by tailoring PLGA’s copolymer ratio, molecular weight, porosity, particle size, and manufacturing conditions [12,13]. 

Naturally occurring chitosan (CS) has the right properties to serve as a polymeric carrier for nanoparticles (NPs) [14]. Biocompatibility, biodegradability, nontoxicity, and low cost all fall into this category. Further, it exhibits bio-adhesive properties and the potential to greatly increase the permeability of hydrophilic compounds [15,16]. To boost chitosan’s mucoadhesive properties, a wide variety of chitosan derivatives have been developed [17]. Because they form covalent bonds with the mucus layer, which are theoretically stronger as compared with the non-covalent bonds, thiol-functionalized polymers have an adhesion advantage over other derivatives [18]. These thiolated polymers (also known as thiomers) interact with cysteine-rich sub-domains of mucus glycoproteins through Disulfide Exchange Mechanisms. This is because Thiolated Chitosan (TCS) is able to form inter- and intra-molecular disulfide bonds at physiological pH, which gives them their in situ gelling capabilities. As a result of the latter process, the carrier matrix is guaranteed to be robust and with integrity [19]. 

Biomimetic HA-blending-AL (HA-AL) nanocrystals were created by Palazzo et al. (2007) as a possible anticancer medication delivery mechanism [11]. Biocompatible and biodegradable poly(lactide-co-glycolide) (PLGA) has been widely employed as a medication delivery carrier [4]. In a rat model of post-menopausal osteoporosis, Sahana et al. (2013) documented the therapeutic benefit of new RIS-HA-loaded NPs over RIS monotherapy for the treatment of osteoporosis [20]. Bilayered mucoadhesive films using risedronate sodium and multicomponent polymers, as reported by Mukherjee et al. It was effectively possible to chemically modify chitosan (thiolation of chitosan) with thioglycolic acid, resulting in increased mucoadhesive qualities, good swelling behavior, and a precise drug release pattern. According to the pharmacological evaluation, the application of thiolated chitosan film containing risedronate was shown to reduce osteoclastic activity [21].

This study proposes the development of nanoconjugates of RIS attached with HA and further conjugate with mPEG for its delivery to the affected bones for the treatment of osteoporosis. Polymer conjugation offers better encapsulation for such a moiety, making the medication more accessible despite its low penetration. The first-step-prepared nanoconjugate was encapsulated in TCS that had been synthesized from CS. In this research, we describe the development of mucoadhesive conjugated nanoparticles, which combine the advantages of small particle size with increased penetration and blood circulation time.

## 2. Materials and Methods

### 2.1. Materials 

Jubilant Life Sciences provided a sample of risedronate sodium (RIS), also known as [1-hydroxy-2-(3-pyridinyl) ethylidene] bis [phosphonic acid] mono-sodium salt hemi-pentahydrate (350.13 g/mol) (Noida, Uttar Pardesh, India). Lipoid is found to be the source of mPEG 2000-DSPE (PE 18:0/18:0-PEG, 2000) (Ludwigshafen, Germany). From S.G. Enterprises, 502.31 g/mol hydroxyapatite (HA) and thioglycolic acid (TGA) 92.12 g/mol were purchased. Dialysis bags (MW 12,000 Da, with 2.5 mm flat width, 16 mm diameter, and 60 mL/ft capacity), chitosan (MWCO: 750,000 Da), sodium tripolyphosphates (TPP), dimethylformamide (DMF) (MWCO: 73.09 g/mol), and DMF were all obtained from Sigma Aldrich, Mumbai, India. Spectro-chem supplied N-hydroxy succinimide (NHS), an Ellman’s reagent, and 1-ethyl-3-(3-dimethylaminopropyl) carbodiimide hydrochloride (EDAC). The other substances and reagents of analytical grade were employed in the study. 

### 2.2. Animals

Wistar rats (225–250 g, both sexes) were used in the ex vivo gut permeation experiment, and the plan for using animals in experimentation was approved by the Jamia Hamdard University, Institutional Animal Ethics Committee (IAEC) (Approval no. 1821/CPCSEA). Lab animals were fed pellets (Lipton, Mumbai, India) and always had access to clean water. The procedures for caring for and using lab animals were followed as per instructions from the National Institute of Health throughout all operations involving animals.

### 2.3. In Silico Molecular Docking

#### 2.3.1. Preparation of Ligand

In this experiment, RIS and RIS-HA-TCS were used as the ligands. The three-dimensional structure of RIS was downloaded in sdf format via PubChem (http://pubchem.ncbi.nlm.nih.gov/). The .SDF file was converted into .pdb file utilizing the software OpenBabel. The ligands.pdb file was converted to a more portable .pdbqt format with the help of Autodock Tools 1.5.6. ChemDraw Professional version 15 was used to create a 3D model of RIS-HA-TCS. 

#### 2.3.2. Preparation of Proteins 

The RCSB (Research Collaboratory for Structural Bioinformatics) protein databank contains the three-dimensional structures of the various molecules of protein. Human farnesyl diphosphate synthase structures were found in the RCSB’s Protein Data Bank (PDB) ID: 1YV5. Water molecules were detected from the enzyme structure using AutoDock tools 1.5.6. Further, Kollman charges and polar hydrogen molecules were added. The final structure was saved in .pdbqt format. 

#### 2.3.3. Docking Analysis Using Autodock Tools 1.5.6

The structures of ligand molecules were inserted into protein structures using AutoDock tools 1.5.6. The complete structures of protein were inserted into the grid box, and the coordinates of the grid box were saved. The flexible ligand molecule was inserted into a rigid protein molecule. Lamarckian genetic algorithm was used to determine the flexible anchoring at the receptor (active site). To determine the energy between the receptor and ligand, calculations were carried out and expressed in Kcal/mol. 

#### 2.3.4. Interaction Plot of Ligand and Protein

The ligand–protein molecule interactions were analyzed using Discovery Studio 2021 version. Different hydrophilic and hydrophobic bonds were expressed by different colors.

### 2.4. Experimental Design

Several independent factors in the creation of nanoparticles were optimized using Design Expert^®^ software (V.13.0; Stat-Ease Inc., Minneapolis, MI, USA). Three centered points were investigated in 15 experimental runs based on a Box–Behnken design. This experimental design was used to observe how different dependent variables were affected by independent variables. ***Y*_1_**: Particle Size; ***Y*_2_**: Polydispersity Index (PDI), and ***Y*_3_**: Percent Entrapment Efficiency were dependent factors, while TPP(A), TCS(B), and Drug−HA(C) were the independent variables, with high (+1), medium (0), and low (−1) values, respectively. All these variables are represented in Table 1.

### 2.5. Synthesis of Thiolated Chitosan (TCS)

**Figure 1:**
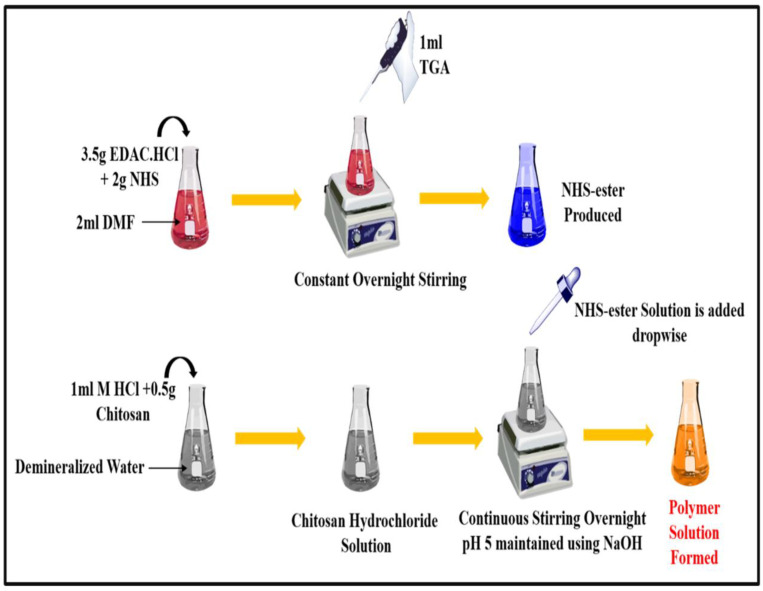
Synthesis of thiolated chitosan (TCS) from chitosan.

TCS was synthesized from chitosan, as depicted in Figure 1, following the method reported and slightly modified [22]. Briefly, 2 mL DMF was added to a flask containing NHS (2 mg), EDAC·HCl (3.5 mg), and TGA (1 mL), and this mixture was constantly stirred overnight. After completing the reaction, reactive NHS-ester was generated. Then, the solution of chitosan hydrochloride was prepared by adding demineralized water to 500 mg of hydrated chitosan in 1 M HCl in a 4 mL flask and shaking it to dissolve the content. After that, the pH of the chitosan hydrochloride solution was adjusted to 5 with 10 M NaOH, and then, the reactive NHS-ester was added drop by drop. After being stirred constantly, this mixture was left for incubation at room temperature for 24 h. Extensive dialyzing in tubing (molecular weight cut-off 12,000 Da; cellulose membrane; dialysis tubing; Sigma Aldrich, India) against HCl (5 mM), which was followed by three cycles of dialyzing against HCl (1 mM) at 8 °C in dark to isolate TCS. Samples and control (chitosan solution) were lyophilized (Labfreez FD-10R, Beijing, China), then kept at 4 °C; this process involved freezing aqueous polymer solutions. Ellman’s reagent technique, FT-IR, DSC were applied to characterize TCS polymer and establish the presence of a thiol group (Perkin–Elmer Spectrum, Mumbai, India).

#### Determination of the Thiol Groups in TCS

- Ellman’s reagent method

The amount of thiol group substitution in the synthesized polymer was measured by spectroscopy using Ellman’s reagent [22]. The symmetric aryl disulfide Ellman’s reagent is highly reactive with the free thiol in the thiol-disulfide interchange. For the preparation of the 2 mg/mL solution, the conjugate compounds and controls were initially dissolved in 5 mm of ultrapure water in 2 mL. Next, 250 µL of aliquots were each given 0.4 mg/mL of DTNB in pH 8 phosphate buffer (0.5 mol/L) and phosphate buffer of pH 8 (0.5 M) of Ellman’s reagent, respectively. At room temperature, the sample was stored for 3 h away from light. To remove any remaining particles, this solution was centrifuged at a speed of 8000 rpm for a period of 20 min. After that, a UV-VIS spectrophotometer was used to detect absorbance at 450 nm (Shimadzu Corp, Kyoto, Japan). Unaltered chitosan was used as a control group.

- Fourier transform infrared spectroscopy technique

Perkin–Elmer Spectrum used the KBr (potassium bromide) method to determine FT-IR spectra of TCS and chitosan. The characteristic peaks present in the newly synthesized polymer (amide bonds and thiol peaks) confirmed the presence of TCS [22].

- Differential scanning calorimetry (DSC)

The thermotropic evaluation of TCS and chitosan was carried out using DSC (Perkin Elmer, Pyris 6, Waltham, MA, USA). In an aluminium crimped pan, about 2 mg of the sample was kept with a lid and subjected to heating between 40 and 400 °C at a scanning rate of 10 °C/min. As a reference, the same empty (blank) pan was used.

### 2.6. Fabrication of RIS-HA Particles

Plain HA particles were mixed with 10 mL of 0.5 mg/mL solution of RIS in distilled water to create RIS-HA particles. For 8 h, the solution was stirred at 37 °C (Table 2). After isolating the deposited phase, it was washed thrice using distilled water and then dried. Drug adsorption on HA was measured, and the particles were conjugated with TCS [4]. Table 2 displays the yields and percent entrapment of RIS with a variety of RIS to HA ratios.

(1) $$Process\;yield=\frac{obtained\;amount\;of\;RIS-HA}{Loaded\;amount\;of\;RIS\;\&\;HA}$$

(2) $$\%EE=\frac{W1-W2}{W1}\times 100$$

where, W1 is the amount of drug in the beginning, W2 is the amount of free/unentrapped drug, and W1 − W2 is the amount of drug entrapped.

### 2.7. Preparation of RIS-HA-TCS Nanoparticles 

The crosslinking agent TPP forms a gel by ionic reaction with the positively charged amino group of TCS [23]. TPP was used to prepare RIS-loaded nanoparticles. All the concentrations of different variables are mentioned in Table 1. TCS (25–35 mg/mL) was briefly dissolved in 2% *w*/*w* CH_3_COOH solution and then allowed to stir overnight at 25 °C. A small amount of NaOH (2 M) solution was added, and the pH of the resulting solution was raised from 4 to 5. After that, RIS-HA (10–20 mg/mL) was added in a 2:1 ratio and vortexed further. The TPP aqueous solution (1–2 mg/mL) was prepared with distilled water and kept in the refrigerator at 0–2 °C for 4 h. At 60 °C, the TCS solution was stirred for 10 min. After transferring the TCS solution to the flask containing the finished RIS-HA, the aqueous TPP solution was added while stirring constantly for 10 min. Once removed from the ice bath, the RIS-HA-TCS nanoparticles were stirred for an additional 15 min to achieve an opalescent suspension. The RIS-HA-TCS nanoparticles were stored in an airtight container for future use after centrifuged at 3000 rpm for 30 min [24].

### 2.8. Optimization of RIS-HA-TCS Nanoparticles

The ideal formulation was chosen for further studies with the goal of having the optimum particle size, the minimum PDI, and the highest possible drug entrapment. In order to reach the set objective, the effect of variables on PDI, particle size, and %EE was analyzed. 

#### Conjugation of RIS-HA-TCS with mPEG

RIS-HA-TCS NPs were further modified with mPEG. Firstly, 100 mg of RIS-HA-TCS NPs were suspended in 20 mL water, and then, after stirring at room temperature overnight, hydroxyl-mPEG-NHS ester (50 mg) was mixed to react with the amino groups on RIS-HA-TCS NPs surface. The required RIS-HA-TCS-mPEG were obtained after centrifugation (5000 rpm) for 10 min; these were then stored in a well-closed container until future use [25].

### 2.9. Characterization of Polymeric Nanoparticles

#### 2.9.1. Particle Size and Polydispersity Index (PDI)

Using Malvern Zetasizer (Malvern Master Sizer 2000, SM, Malvern, UK), laser light scattering was used to determine the particle size of both the optimized formulation RIS-HA-TCS and RIS-HA-TCS-mPEG nanoparticles. After diluting with distilled water, RIS-HA-TCS and RIS-HA-TCS-mPEG nanoparticles were mixed in the sample unit. The experiments were carried out three times (*n* = 3).

#### 2.9.2. Determination of Drug Entrapment Efficiency (%EE) 

The entrapment efficiency of RIS in the nanoparticles was determined indirectly by determining the free or unentrapped RIS present in the optimized formulation after centrifugation. In this process, the optimized formulation was centrifuged at 15,000 rpm for 30 min at 4 °C. A UV-visible spectrophotometer (Shimadzu, Model UV-1601, Kyoto, Japan) was used to measure the concentration of RIS in the supernatant that was taken in the tube after it had been separated and diluted 10 times with distilled water and set to 263 nm. Therefore, the following calculation was used to determine the EE as a percentage. The experiment was conducted three times, and the results were presented as mean value ± standard deviation.

(3) $$\%EE=\frac{W1-W2}{W1}\times 100$$

where, W1 is the amount of drug in the beginning, W2 is the amount of free/unentrapped drug, and W1 − W2 is the amount of drug entrapped.

#### 2.9.3. Transmission Electron Microscopy (TEM)

Transmission electron microscopy (Morgagni 268D-SEI, Thermofisher, Bleiswijk, The Netherlands) operated at 100 kV with point-to-point resolution was used to examine the morphology of RIS-HA-TCS and RIS-HA-TCS-mPEG nanoparticles. Negatively dyed with phosphotungstic acid, the RIS-HA-TCS and RIS-HA-TCS-mPEG nanoparticles were diluted 50-fold in double-distilled water and dried on the carbon-coated grid for examination.

#### 2.9.4. Differential Scanning Calorimetry (DSC)

The thermotropic properties of RIS, HA, mPEG, a physical mixture of RIS with excipients, and lyophilized optimized RIS-HA-TCS and RIS-HA-TCS-mPEG nanoparticles were observed using DSC (Pyris 6, Perkin Elmer, Waltham, MA, USA). The samples were placed in an aluminum pan and subjected to heating between 40 and 400 °C at a scanning rate of 10 °C/min. 

#### 2.9.5. Fourier Transform Infrared Spectroscopy (FTIR)

Lyophilized optimized RIS-HA-TCS and RIS-HA-TCS-mPEG nanoparticles, as well as FTIR spectra of RIS, HA, mPEG, surfactant, and physical mixtures of RIS with excipients, were recorded (Bruker Optik GmbH, Ettlingen, Germany). The scanning range was adjusted from 4000 to 400 cm^−1^ with a resolution of 4 cm^−1^.

### 2.10. In Vitro Release Study 

The dialysis bag (12,000 g/mol:MW and 16 mm: diameter) method was used in the drug release experiment [26]. The dialysis bag was immersed in simulated intestinal fluid, pH 6.8, for pre-treatment and kept for 24 h before the experiment began. To dissolve the optimal formulation of 5 mg RIS, a dialysis sac was submerged in 500 mL of freshly produced SIF at 37 °C in a dissolving flask. Samples of 2 mL were taken at regular intervals (15 min, 30 min, 1, 2, 4, 6, 8, 12, and 24 h) while the digital magnetic stirrer was running at 100 rpm. To keep the sink state, an equal amount of SIF was refilled. Finally, a UV-VIS spectrophotometer set to 263 nm was used to determine the exact amount of RIS in each sample. The release of RIS from optimized RIS-HA-TCS and RIS-HA-TCS-mPEG nanoparticles was compared with the RIS suspension and commercially available preparation (RISOSFOS 35 mg/Week). The study was performed thrice (*n* = 3).

### 2.11. Ex Vivo Intestinal Permeation Study 

A modified version of the everted sac model was used to examine the passage of RIS through the intestinal barrier [27,28]. Following overnight fasting, animals were administered diethyl ether anaesthesia before being sacrificed via cervical dislocation. After surgically removing the small intestine, a 5-cm portion was carved out, and the food remnants were washed away in normal saline. After everting the intestine with a glass rod, 2 ml of Krebs–Ringer solution was injected. In Krebs–Ringer solution (50 mL), the portion of the intestine sac containing 2000 μg RIS was kept, and the entire setup was maintained at 37 ± 0.5 °C, aerated with O_2_ (95%) and CO_2_ (5%). At 0, 15, 30, 45, 60, 75, and 90 min, 2 ml aliquots of serosal medium were obtained for quantification of RIS permeated. A UV-VIS spectrophotometer calibrated to a wavelength of 263 nm was used to measure the amount of RIS that passed through the gut. Filtration of the sample was performed through a syringe filter (0.45 µm pore size) before analysis. For the optimized RIS-HA-TCS, RIS-HA-TCS-mPEG nanoparticles, and commercial formulation, a similar experiment was carried out. The below-given formula was used for calculating the Apparent Permeability (Papp) coefficient of RIS suspension, marketed formulation, optimized RIS-HA-TCS, and RIS-HA-TCS-mPEG nanoparticles:

(4) Papp=FA×C0 cm min−1

where, F is permeation flux, C_0_ is concentration at outset, and A is ileum’s total surface area.

## 3. Results

### 3.1. In Silico Activity

**Figure 2:**
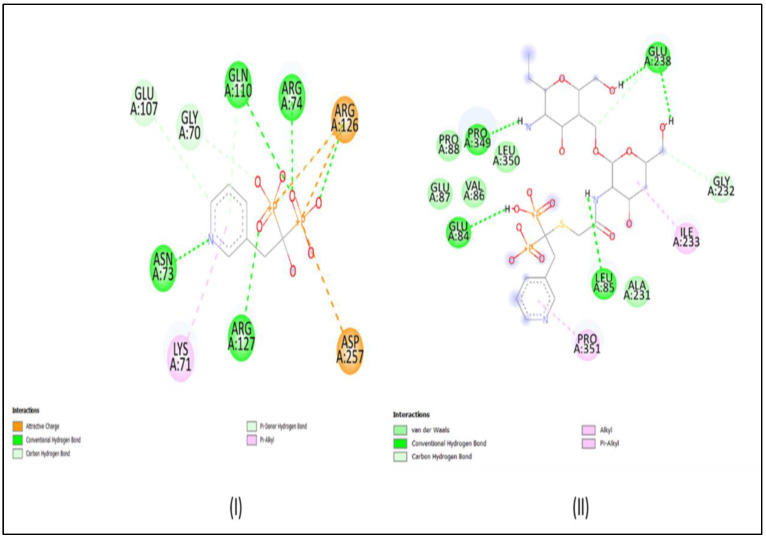
Docking results: (**I**) RIS, and (**II**) RIS-HA-TCS.

AutoDock Vina was used to carry out the docking studies of RIS and conjugated RIS for farnesyl pyrophosphate synthetase enzyme. Binding energies below 5 kcal/mol represent weak binding, whereas higher values, above 10 kcal/mol, signify strong binding. Furthermore, the protein−ligand interaction structures were obtained using Discovery Studio and they are depicted in Figure 2. The binding affinity for RIS and RIS-TCS-HA were found to be −6.86 and −27.70 kcal/mol, respectively. Moreover, number of hydrogen bonds for both RIS and RIS-TCS-HA was found to be five. These results showed that the formulation that we prepared in our study had four times better binding than RIS alone.

### 3.2. Synthesis of Thiolated Chitosan (TCS)

**Figure 3:**
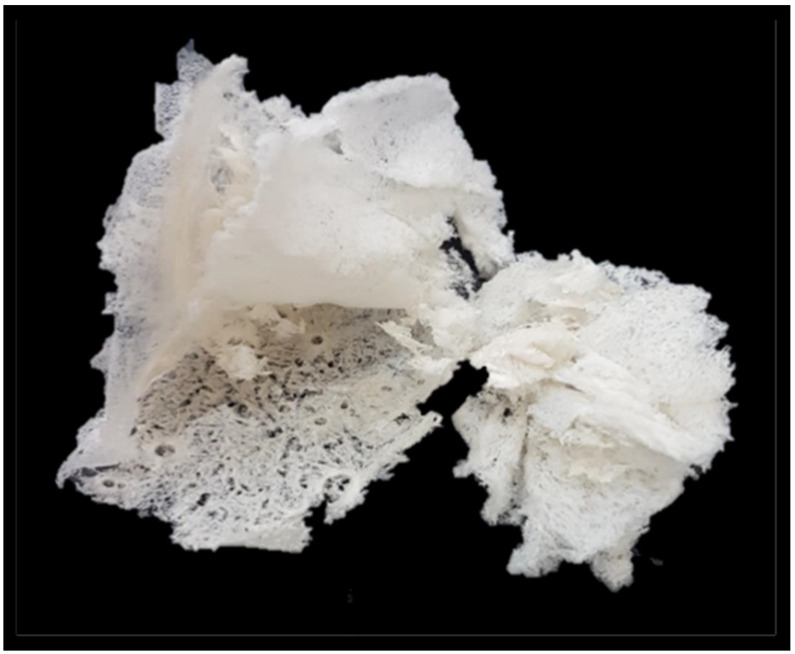
Structure of the synthesized fibrous thiolated chitosan (TCS).

The TCS synthesized by the above method was found to be more significant than alternative methods [29]. We chose DMF as the reaction medium instead of water as compared to the previously published studies. First, the reactive NHS-ester, which was discovered to be more reliable and stable for the subsequent reaction, was synthesized [30]. Next, the cationic chitosan polymer’s main amino groups were coupled with the reactive NHS-ester. These modifications were made (a) to prevent unstable Oacylisourea ester hydrolyzing in H_2_O and (b) it is possible that the concentration of the target reactant may rise if the reactive NHS-ester was to be produced. As shown in Figure 3, TCS appeared as white, fibrous in structure and was odourless. TCS was also soluble in an aqueous medium.

### 3.3. Determination of the Thiol Groups in TCS 

#### 3.3.1. Ellman’s Method

The thiol group immobilization by the polymer was found to be 2402.23 ± 2.71 μmol/g.

#### 3.3.2. Fourier Transform Infrared Spectroscopy (FT-IR)

**Figure 4:**
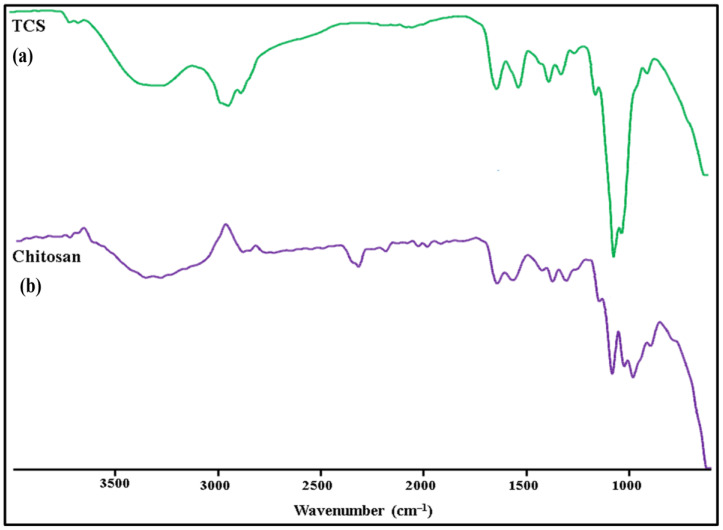
FTIR spectra: (**a**) TCS and (**b**) Chitosan.

In Figure 4, the FT-IR spectra of TCS and chitosan are shown. The following distinctive peaks were observed in the chitosan spectrum: (a) 3410 cm^−1^ due to O-H and N-H, (b) 2924 cm^−1^ due to C-H,1623 cm^−1^, (c) 1513cm^−1^ due to N–H, (d) 1088 cm^−1^ due to C–N, (e) 1380 cm^−1^ (C-H), (f) 651 cm^−1^ (NH_2_), (g) 1248cm^−1^ (O-H), (h) 1153cm^−1^ (C-O-C). In TCS, all characteristic peaks were observed except NH_2_ peaks. Additional peaks of the newly created -CONH_2_ bond were also found: amide band I at 1629 cm^−1^, amide band at 1524 cm^−1^, and thiol group peaks at 1251 cm^−1^ due to NH_2_ reaction between chitosan and carboxyl groups of TGA [31]. 

#### 3.3.3. Differential Scanning Calorimetry (DSC)

**Figure 5:**
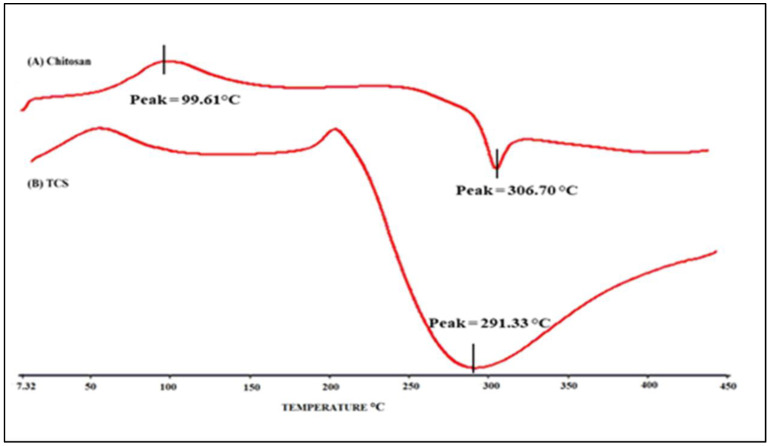
DSC thermogram: (**A**) Chitosan and (**B**) TCS.

Figure 5 illustrates the DSC thermogram of chitosan and TCS. As evident from the thermogram, an endothermic peak was observed at 98.95 °C and an exothermic peak was observed at 306.876 °C in a chitosan sample, and a peak at 217.2 °C was observed in TCS thermogram. A peak at 217.2 °C was observed due to TCS, which was formed when the chitosan was crosslinked with TGA. As evident from the DSC of chitosan, this peak was absent. The crosslinking of chitosan with TGA [32], which was not present in chitosan, is indicated by the considerable change in the peak and endothermic enthalpy values.

### 3.4. Fabrication of RIS-HA Particles

The nanoparticles prepared by combining RIS with HA in different ratios showed different entrapment values. Among all the different ratios, the RIS:HA ratio of 2:1 was finally selected for further studies as it had shown higher drug entrapment value (93.97% ± 1.56) and sufficient yield value (74.80% ± 2.61).

### 3.5. Experimental Design Optimization

**Figure 6:**
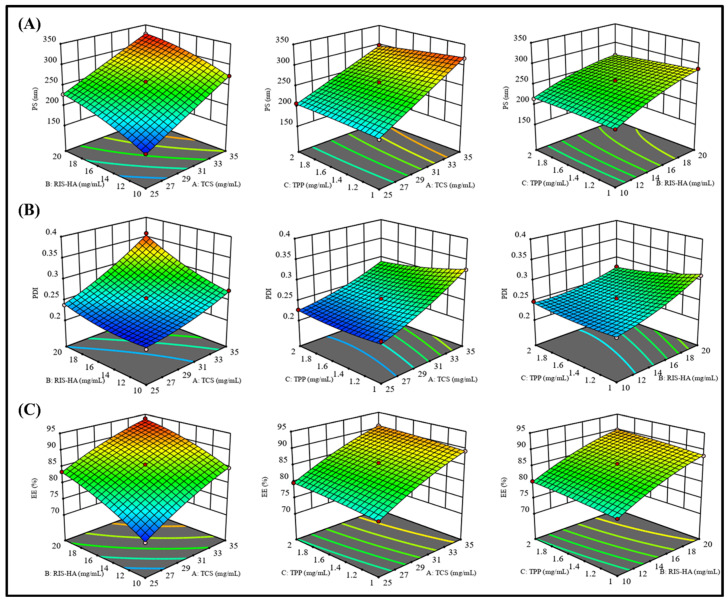
Response surface curve: showing the effect of factors on dependent variables: (**A**) Particle size (nm), (**B**) PDI, and (**C**) Entrapment efficiency (%) of RIS-HA-TCS nanoparticles within Box–Behnken statistical design.

Box–Behnken statistical design (BBD) was used to formulate and optimize the RIS-HA-TCS nanoparticles in which the influence of different variables on the responses was observed simultaneously [26]. Here, the impact of independent variables: (A) TPP, (B) TCS, and (C) Drug−HA on the dependent variables (*Y*_1_) Particle size, (*Y*_2_) Polydispersity index (PDI), and (*Y*_3_) Entrapment efficiency (EE) was observed as shown in Table 3. As depicted in Table 4, for all the response parameters the Predicted R^2^ values and the Adjusted R^2^ values were in a reasonable agreement. To be in a reasonable agreement, the Predicted R^2^ and Adjusted R^2^ values must be within around 0.20 of one another [33]. The quadratic model was shown to have a low coefficient of variance across all responses, indicating its viability for use in design space exploration. Table 4 provides a summary of the polynomial equations for the dependent variables *Y*_1_*, Y*_2_, *Y*_3_. Positive signs in the polynomial equation signified a direct correlation between the factor and responses (dependent variables), whereas negative signs represented an antagonistic connection between factors and responses. Figure 6 shows response surface graphs.

#### 3.5.1. The Effect of Independent Variables on Particle Size (Y_1_)

As polymer (TCS), crosslinking agent (TPP), and Drug−HA complex concentrations were increased, particle size was shown to decrease. One possible explanation for this finding is that the polymeric layer shrank because of increased crosslinking between positively charged amino groups in TCS and the PO^4−^ ions in TPP [34]. 

#### 3.5.2. The Effect of Independent Variables on PDI (Y_2_)

Table 3 displays that the PDI value for all the produced nanoparticles was less than 0.5, indicating a smaller size distribution [35].

#### 3.5.3. The Effect of Independent Variables on Encapsulation Efficiency (Y_3_)

The effects of independent factors on %EE were explained using response surface plots, such as the one in Figure 6, to explain the impact of factors on the encapsulation efficiency. Due to the crosslinking of the TCS polymer and TPP as crosslinking agent, a decrease in the amount of drug leakage was observed, but it caused nanoparticles to develop a rigid structure [36]. These findings corroborated with an earlier study [37], where it was found that greater drug entrapment efficiency was associated with the development of higher disulfide bonds between TCS and PO^4-^ ion crosslinking.

### 3.6. Selection of Optimized RIS-HA-TCS Nanoparticles

Using the mathematical optimization technique implemented in Design Expert^®^ software (V.13.0; Stat-Ease Inc., Minneapolis, MN, USA), the RIS-HA-TCS nanoparticles with the highest % EE and the smallest particle size and PDI were selected. The formulation containing 30 mg/mL of TCS, 15 mg/mL of Drug−HA, and 1.5 mg/mL of TPP was created to fulfill the criterion of optimized formulation after “trading off” distinct responses with statistical desirability function. The optimized RIS-HA-TCS nanoparticles (RUN 6) exhibited a particle size of 252.1 ± 2.44 nm, 0.11 ± 0.01 PDI, and %EE of 85.4 ± 2.21%.

The optimized formulation was found to be an opalescent solution. 

The formulation without mPEG was termed RIS-HA-TCS, and the formulation containing mPEG was represented as RIS-HA-TCS-mPEG. mPEG was used to increase the drug’s half-life and stability. The particle size of RIS-HA-TCS-mPEG formulation was significantly higher than RIS-HA-TCS formulation, but its PDI was lower than RIS-HA-TCS, which indicated the mono-dispersity of particles. The entrapment efficiency of RIS-HA-TCS-mPEG was high as compared to RIS-HA-TCS. 

### 3.7. Characterization of RIS-HA-TCS and RIS-HA-TCS-mPEG

#### 3.7.1. Particle Size and Polydispersity Index

**Figure 7:**
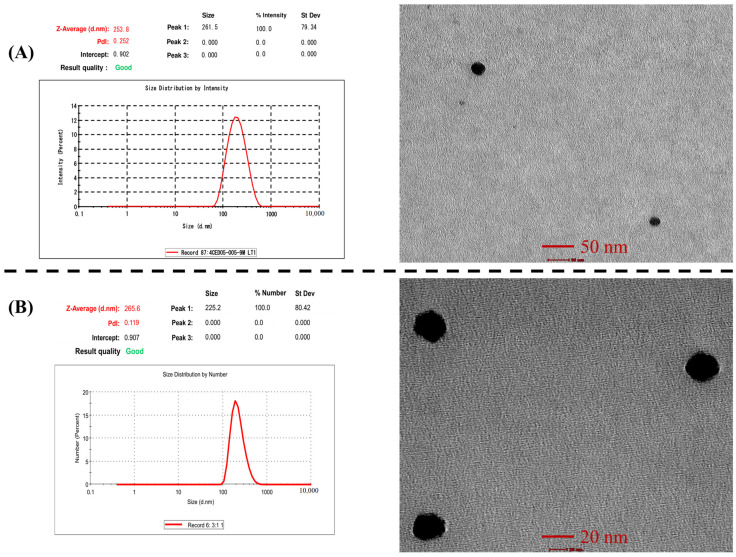
Image represents: (**A**) particle size and TEM of RIS-HA-TCS and (**B**) Particle size and TEM of RIS-HA-TCS-mPEG.

The particle size of RIS-HA-TCS and RIS-HA-TCS-mPEG nanoparticles was found to be 252.1 ± 2.44 and 264.9 ± 1.91 nm, respectively, whereas PDI of RIS-HA-TCS and RIS-HA-TCS-mPEG were 0.2 ± 0.01 and 0.120 ± 0.01, which indicated a mono-dispersed system i.e., RIS-HA-TCS and RIS-HA-TCS-mPEG nanoparticles were found to be uniformly dispersed in the entire formulation. Both formulations’ particle sizes were appropriate for oral drug administration. The PDI value closer to zero showed that the RIS-HA-TCS-mPEG formulation was more homogeneous. These results are demonstrated in Figure 7.

#### 3.7.2. Entrapment Efficiency (%EE)

The entrapment efficiency of RIS-HA-TCS and RIS-HA-TCS-mPEG nanoparticles was found to be 85.4 ± 2.21% and 91.1 ± 1.17%, respectively. An increase in TCS, TPP, and Drug−HA concentrations was associated with an increase in EE percentages. This is because the TCS polymer crosslinked with TPP formed nanoparticles with a hard structure, reducing the drug leakage [36]. 

#### 3.7.3. Transmission Electron Microscopy Analysis 

Using a transmission electron microscope, the shape morphology of RIS-HA-TCS and RIS-HA-TCS-mPEG nanoparticles was observed. The TEM exhibited a spherical shape, uniformly dispersed and non-aggregated NPs. The spherical nanoparticles showed that TCS and TPP were successfully crosslinked. Specifically, the ionic gelation that gave the nanoparticles their structural stability was caused by the crosslinking between negatively charged phosphate ions of TPP and positively charged amine groups of TCS [38]. These results are demonstrated in Figure 7.

#### 3.7.4. Differential Scanning Calorimetry 

**Figure 8:**
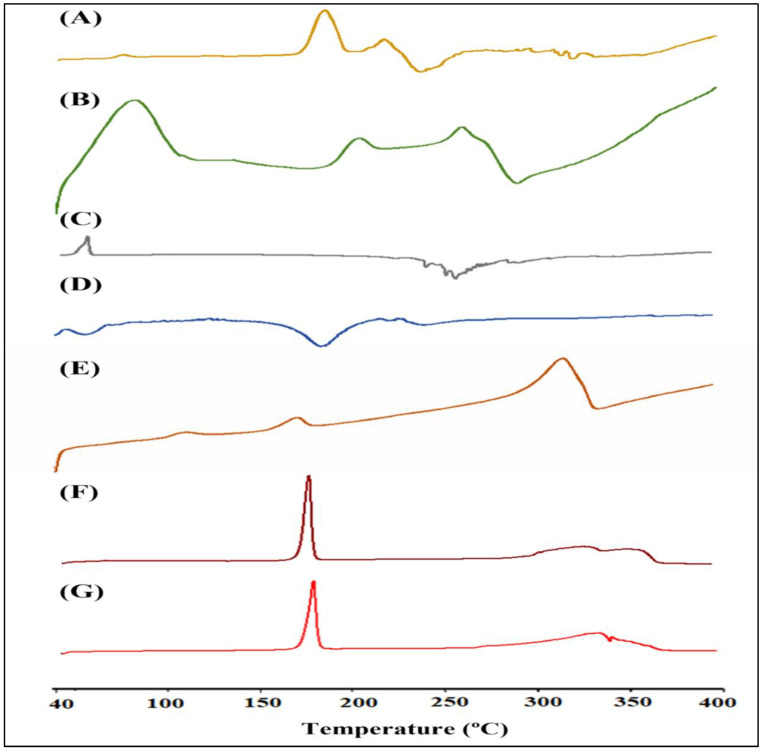
DSC thermogram: (**A**) RIS, (**B**) HA, (**C**) mPEG, (**D**) Physical mixture, (**E**) Fabricated RIS-HA, (**F**) Optimized RIS-HA-TCS, and (**G**) RIS-HA-TCS-mPEG.

The DSC thermogram of RIS, HA, mPEG, a physical mixture of excipients with drug, fabricated RIS-HA, and optimized RIS-HA-TCS and RIS-HA-TCS-mPEG nanoparticles are represented in Figure 8. As evident, the thermogram showed that RIS had two endothermic peaks at 205 °C and 245 °C corresponding to the solvent loss. An exothermic peak at 265 °C corresponded to the melting point of the drug [21]. As evident from the published literature, pure mPEG showed a distinct phase transition at around 40 °C, and its degradation peaks are observed at around 280 °C [39,40]. The endothermic peak of the HA DSC thermogram occurred at approximately 60 °C, perhaps as a result of moisture loss, while the endothermic peak occurred at approximately 270 °C, which was likely a result of HA degradation. As the melting point of HA is around 1100 °C, it was not captured in DSC [41]. At the same temperature range, the DSC of drug–excipient showed that the primary peaks of the RIS and excipients were evident, and there was no interaction between them. The RIS-HA-TCS and RIS-HA-TCS-mPEG nanoparticles showed no sharp endothermic peak of RIS because RIS was fully incorporated and molecularly distributed in the solid matrix in an amorphous state. An endothermic peak at a melting point of 164.5 °C corresponds to the mannitol (cryoprotectant) and was the only peak observed in nanoparticles [42].

#### 3.7.5. Fourier Transform Infrared Spectroscopy (FTIR) Spectra

**Figure 9:**
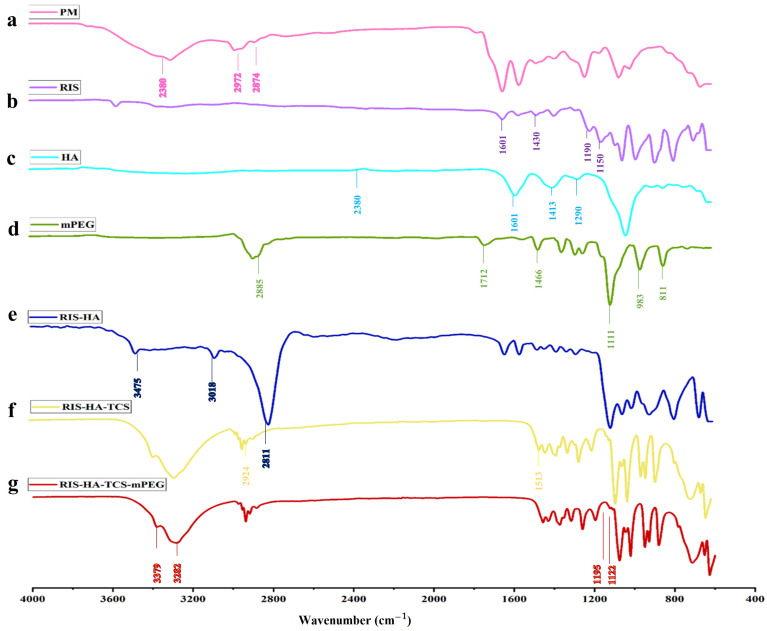
FTIR spectra for (**a**) PM, (**b**) RIS, (**c**) HA, (**d**) mPEG, (**e**) RIS-HA, (**f**) RIS-HA-TCS, and (**g**) RIS-HA-TCS-mPEG.

FTIR Spectra overlay of RIS, HA, TCS, and mPEG are shown in Figure 9. The following peaks for RIS were observed (a) 1150 cm^−1^ due to an aliphatic P-O stretch, (b) Aromatic P-O stretch is at 1190 cm^−1^, while aromatic C-H stretch is at 3080–3010 cm^−1^, (c) O-H Stretch at 3609–3329 cm^−1^, and (d) Stretching from C-C, 1601 cm^−1^, and from C-N, 1430 cm^−1^ [21]. The spectra of TCS showed the following distinctive peaks: (a) 3410 cm^−1^ due to O–H and N–H, (b) 2924 cm^−1^ due to C–H, (c) 1513 cm^−1^ due to N–H, (d) 1088 cm^−1^ due to C–N, (e) 1380 cm^−1^ due to C–H, (f) 1248 cm^−1^ due to O–H, (g) 1153 cm^−1^ (C-O-C), and (h) 1629 cm^−1^ (amide band I), (i) 1524 cm^−1^ (amid band II) and thiol groups represented by 1251 cm^−1^ peak [31]. HA was found to have a peak between 2400 and 1600 cm^−1^ (O=P-H stretching) [4]. Alkane (C-H), C=C and C=0 stretch, frequencies of methoxy polyethylene glycol molecule are responsible for the 2885, 1712, 1466, and 1111 cm^−1^ peaks [43]. All the respective peaks for RIS, HA, TCS, and mPEG were also observed in the formulation.

### 3.8. In Vitro Drug Release Experiment

**Figure 10:**
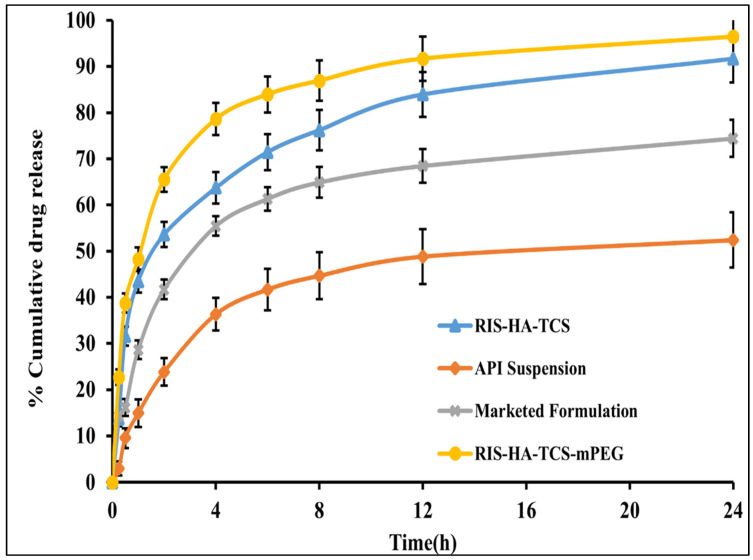
In vitro drug release profile of RIS from API suspension, marketed preparation, RIS-HA-TCS and RIS-HA-TCS-mPEG in SIF pH 6.8.

For the purpose of measuring RIS release in vitro, the dynamic dialysis approach, which is often used to keep nanoparticles from leaking into the dissolving medium, was implemented [44]. In vitro experiments were performed in SIF (pH 6.8) for the optimized RIS-HA-TCS, RIS-HA-TCS-mPEG, API suspension, and marketed formulation Figure 10. The drug release in the first 2 h after administering RIS-HA-TCS and RIS-HA-TCS-mPEG were found to be 52.32 ± 2.72% and 66.13 ± 2.52%, respectively, followed by a slow drug release for the next 24 h (91.74 ± 5.14% and 95.13 ± 4.64%, respectively). Under similar experimental conditions, pure API suspension and commercialized formulation demonstrated 56.11 ± 5.19% and 74.69 ± 3.98% drug release over a period of 24 h when tested for in vitro release. With an increase in polymer content, more nanoparticles were retained by the intestinal tissue, as determined by the in vitro drug release research. These findings may be attributed to the enhanced release of drug from nanoparticles firmly adhered to the mucous layer through covalent linkage due to the presence of increased TCS content [29]. These results were in good agreement with Zhou [45], who discovered that the oral administration of insulin thiolated nanoparticles improved insulin adherence to the mucosal membrane. 

### 3.9. Ex Vivo Intestinal Permeation Study 

**Figure 11:**
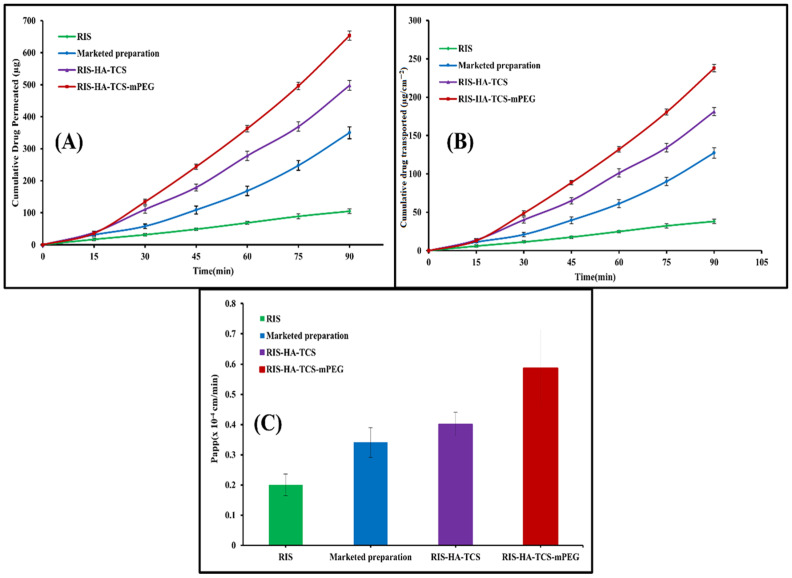
Intestinal permeation study result showing: (**A**) Cumulative amount of drug permeated (µg) vs time (min), (**B**) cumulative amount of drug transported (µgcm^−2^) vs. time (min), (**C**) P_app_ of RIS, marketed formulation, RIS-HA-TCS and RIS-HA-TCS-mPEG.

The gut permeability of RIS was determined using a non-everted gut permeation investigation using nanoparticles, a drug suspension, and a commercially available preparation. This research explains how drugs are absorbed by the body once they reach the digestive tract. In Figure 11, the intestinal penetration profile of RIS from the optimized RIS-HA-TCS, the RIS-HA-TCS-mPEG, RIS suspension, and the commercially available preparation is shown. Papp of the RIS-HA-TCS-mPEG nanoparticles was found to be 0.5858 ± 0.1227 × 10^−4^ cm/min, which was higher in comparison with RIS-HA-TCS formulation 0.4011 ± 0.03938 × 10^−4^ cm/min, marketed preparation (0.3401 ± 0.04912 × 10^−4^ cm/min) and RIS suspension (0.2005 ± 0.03599 × 10^−4^ cm/min). Because nanoparticles diffuse more quickly across the gut membrane [46], their tiny size and total internalization of RIS into nanoparticles were attributed to the significantly improved release of RIS by RIS-HA-TCS-mPEG and RIS-HA-TCS.

## 4. Conclusions

In conclusion, the successfully prepared and optimized RIS-HA-TCS nanoparticles, employing the ionic gelation method and Box–Behnken design, demonstrate promising attributes for the oral treatment of osteoporosis. The PEGylation of these nanoparticles further enhances their stability and drug half-life. Comparative analysis with RIS-HA-TCS reveals that RIS-HA-TCS-mPEG nanoparticles exhibit superior particle size and entrapment efficiency. Additionally, PEGylated nanoparticles display improved in vitro drug release and ex vivo permeability. The notable impact of thiolated chitosan on drug release rate underscores its significance as a carrier for controlled oral drug administration. While presenting a compelling approach, further investigations into pharmacokinetics, histopathology, and pharmacodynamics are essential to validate the potential of RIS-TCS-HA, with or without mPEG, as a novel oral therapeutic strategy for osteoporosis.

## Figures and Tables

**Table 1:** Various variables (independent and dependent) used for the preparation and optimization of RIS-HA-TCS nanoparticles in the Box–Behnken design (BBD).

Variables	Constraints		
**Independent**	+1	0	−1
**A = TPP (mg/mL)**	2	1.5	1
**B = TCS (mg/mL)**	35	30	25
**C = Drug − HA (mg/mL)**	20	15	10
**Dependent**	**Objectives**		
***Y*** **_1_** ** = particle size (nm)**	Optimum		
***Y*_2_ = PDI**	Minimize		
***Y*** **_3_** ** = EE (%)**	Maximize		

**Table 2:** Yield of product and entrapment efficiency of RIS-HA particles.

S.NO.	RIS:HA	Process Yield	RIS %EE
1	1:1	73.27 + 4.37	70.30 + 1.01%
2	1:2	74.80 + 2.61	73.87 + 0.72%
3	1:3	84.57 + 3.06	62.27 + 1.27%
4	1:4	70.83 + 4.93	87.23 + 0.91%
5	1:5	86.07 + 3.19	84.30 + 0.70%
6	2:1	60.87 + 6.62	93.97 + 1.56%
7	3:1	75.17 + 5.33	59.00 + 1.28%
8	4:1	79.90 + 7.81	76.14 + 1.15%
9	5:1	65.33 + 2.96	69.33 + 1.08%

**Table 3:** The Box–Behnken experimental design of polymeric nanoparticles (RIS-HA-TCS) and evaluated response parameters (*n* = 3).

Run	A: TCS	B: RIS-HA	C: TPP	Particle Size	PDI	EE
	(mg/mL)	(mg/mL)	(mg/mL)	(nm)		(%)
**1**	30	15	1.5	259.63	0.255	85.37
**2**	30	15	1.5	256.46	0.252	85.42
**3**	30	20	1	288.23	0.312	88.14
**4**	25	15	2	207.38	0.227	79.66
**5**	30	10	2	214.77	0.247	80.31
**6**	30	15	1.5	253.82	0.252	85.73
**7**	35	15	2	298.16	0.283	90.67
**8**	25	10	1.5	163.65	0.214	71.92
**9**	25	15	1	198.21	0.231	77.68
**10**	25	20	1.5	228.24	0.238	83.47
**11**	35	20	1.5	326.38	0.359	93.65
**12**	35	10	1.5	273.28	0.273	84.68
**13**	30	10	1	217.23	0.238	78.34
**14**	35	15	1	317.17	0.326	89.23
**15**	30	20	2	267.12	0.275	89.83

**Table 4:** Regression analysis parameters for the responses *Y*_1_, *Y*_2_, and *Y*_3_.

Models	R^2^	R^2^ (Adjusted)	R^2^ (Predicted)	S.D.	C.V. (%)
**Response, *Y*_1_**					
Linear	0.9786	0.9728	0.9577	7.61	–
2F1	0.9893	0.9813	0.9574	6.31	–
Quadratic	0.9985	0.9958	0.9837	3.00	1.19
**Response, *Y*_2_**					
Linear	0.8829	0.8509	0.7699	0.0155	–
2F1	0.9654	0.9394	0.9026	0.0099	–
Quadratic	0.9928	0.9797	0.8877	0.0057	2.16
**Response, *Y*_3_**					
Linear	0.9799	0.9744	0.9667	0.92	–
2F1	0.9836	0.9713	0.9537	0.98	–
Quadratic	0.9994	0.9983	0.9927	0.23	0.28

*Y*_1_ = + 256.64 + 52.19 A + 30.13 B − 4.18 C − 2.87 AB − 7.04 AC − 4.66 BC − 0.1783 A^2^ − 8.57 B^2^ − 1.23 C^2^. *Y*_2_ = + 0.2530 + 0.0414 A + 0.0265 B − 0.0094 C + 0.00155 AB − 0.0098 AC − 0.0115 BC + 0.0084 A^2^ + 0.0096 B^2^ + 0.0054 C^2^. *Y*_3_ = + 85.51 + 5.69 A + 4.98 B + 0.8850 C − 0.6450 AB − 0.1350 AC −0.0700 BC − 0.9608 A^2^ − 1.12 B^2^ − 0.2358 C^2^. R^2^ = coefficient of correlation; S.D.= standard deviation; C.V. = Coefficient of Variation.

## Data Availability

Data are contained within the article.

## Abbreviations

BPs: Bisphosphonates, NPs: Nanoparticles, CS: Chitosan, FPPS: farnesyl pyrophosphate synthase, %EE: Percentage entrapment efficiency, PDI: Polydispersity index HA: Hydroxyapatite, PLGA: Poly(lactide-co-glycolide), RIS: Risedronate, SERM: Selective estrogen receptor modulators, TCS: Thiolated chitosan.
